# Infant and young child feeding practices and the factors that influence them: a qualitative study

**DOI:** 10.1186/s41043-023-00371-9

**Published:** 2023-04-13

**Authors:** Mary Beth Weber, Wendy Palmer, Monica Griffin, Jean A. Welsh

**Affiliations:** 1 Hubert Department of Global Health, Emory University, 1518 Clifton Road, NE, Atlanta, GA USA; 2 Children’s Healthcare of Atlanta, Atlanta, GA USA; 3 Department of Pediatrics, School of Medicine, Emory University, Atlanta, GA USA; 4 Emory Global Diabetes Research Center, Emory University, Atlanta, GA USA

**Keywords:** Early child feeding, Maternal behavior, Nutrition

## Abstract

**Background:**

Early child feeding is important for healthy growth and forming positive eating behaviors.

**Methods:**

This qualitative study sought to describe early childhood feeding behaviors, challenges, and opportunities through four focus group discussions with a diverse group of mothers of at least one child under two years or pregnant with their first child.

**Results:**

Although providing healthy foods was a priority, feeding behaviors reflected the mothers’ partial understanding of infant and child nutrition. Mothers sought guidance on early child feeding from several sources, including in-person and virtual relationships but made decisions based largely on their own instincts. Participants consulted clinicians the least often, and mothers often felt frustrated by strict guidelines and negative messaging. Mothers were most receptive to suggestions when they felt supported and valued in the decision-making process.

**Conclusions:**

In order to help mothers provide the best nutrition for their young children, clinicians should use positive tones, provide flexibility when possible, and work to create open lines of communication with parents.

## Introduction

Despite increased efforts to reverse rising trends in child obesity, 18.5% of US adolescents are obese [1], the most since concern about the epidemic was first highlighted [2]. Children who are overweight at age five are four times as likely as their normal-weight peers to become obese later in childhood [3]. The paucity of evidence on interventions effective in improving weight and reversing obesity [4] points to the need for greater focus on prevention, beginning very early in childhood. Efforts to do so are having some success; among children participating in the Supplemental Nutrition Program for Women, Infants, and Children (WIC), the prevalence of obesity decreased from 15.9% in 2010 to 14.5% in 2014 [5].

Early feeding practices such as breastfeeding, a varied diet [6], and delayed introduction of juice and other sugary beverages [7] influence later obesity risk. What a young child eats is directly dependent on the knowledge, perceptions, and practices of their parents and other caregivers, yet little is known about how to modify these feeding practices [8]. This study: explores maternal knowledge, beliefs, and behaviors regarding early child feeding; identifies sources of information and other factors that influence mothers’ feeding practices; and reports on how to improve early feeding information provided by clinicians.

## Methods

### Participants

Focus group discussions included English-speaking, Caucasian, African American or Hispanic women living in metropolitan Atlanta, GA; pregnant with their first child or a mother of at least one child aged 0–2 years; responsible for most child feeding decisions in their household; interested in health and nutrition; aged 21–34 years; with some high school or greater education and a household income of $100,000 per year or less; open to healthcare messaging; and not working in healthcare or health marketing. Recruitment targeted women with a mix of marital statuses. A marketing research company conducted recruitment and data collection.

### Data collection

Focus group discussions were held on March 9 and 10, 2016. Each group included a mix of first-time expectant mothers, first-time mothers of children 0–2 years, and mothers of children 0–2 years with at least one older child. Groups were stratified by income (low income = annual household income of $35,000 or less/WIC eligible or middle income = annual household income of $36,000-$100,000) and race/ethnicity (African American or Caucasian and Hispanic), with one group each per income/race-ethnicity category. Focus group discussions were led by an experienced moderator from the marketing research company and lasted approximately 90 min. The moderator used a semi-structured interview guide to explore participants’ child feeding practices including food choices and meal behaviors, attitudes and perceptions of mealtime, sources of nutrition information, and what and who influences food choices.

The study was approved by the Emory University Institutional Review Board (#00115205). This secondary analysis used only de-identified data, namely verbatim transcripts created from recordings of the focus group discussion. Discussions were video-recorded, but the videos were blurred, so individual participants were not identifiable. Participants provided verbal consent to group participation and recording of the discussion. Study data are available from the authors upon reasonable request.

### Data analysis

De-identified, verbatim transcripts were loaded into MaxQDA (Verbi Software, Germany) for data management and manipulation. The textual data were reviewed through a close reading of the transcripts, and an initial code system was developed. The code system was refined through application of codes to two groups and a discussion with the research team. The updated code system was then applied to a subset of the data by two researchers, and the coded sections were compared. All areas of discrepancy were discussed, and the code system was finalized. The final code system was applied to the data, and a thematic analysis was conducted wherein thick descriptions of key themes around early child feeding practices and views on nutrition information sources were developed.

## Results

Herein, we describe the perceptions, beliefs, and experiences of the 22 participants in regard to how they fed (or planned to feed) their infants and young children around the following thematic areas: beliefs about healthy eating, common feeding practices, drivers of food choice, and suggestions for improving early feeding education and resources offered by clinicians.

### Beliefs about healthy eating

Across groups, women described home cooked foods, fresh produce, and certain cooking methods (grilling, baking, steaming or boiling) as being healthy. Many mothers also talked about the need to have a balanced diet and meals including items “from each food group…making it colorful, at least two vegetables, protein, a little starch.” Mothers in two groups felt that organic foods and certain “superfoods” (e.g., avocado) were particularly healthy. On the other hand, mothers agreed that processed foods (e.g., chicken nuggets, pizza rolls), sugary, fried, or fatty foods, Southern/Soul foods, pasta, produce that is not fresh, and an unvaried diet were unhealthy.

### Common feeding practices

#### Strategies for encouraging healthy eating

Participants described several methods to help their children eat healthier including swapping less healthy foods for something they viewed as more nutritious. For example, one mother takes the cheese off pizza, another participant swapped fried chicken nuggets for grilled at a fast food restaurant, and another mother served sliced, fried ham in place of bacon. Several mothers fib to their small children about these food swaps, for example by saying frozen yogurt is ice cream or giving their toddler raisins in an M&M bag. Mothers justify these untruths as follows: “When he gets older he’s going to be so used to the habit that he’s not going to care anymore, he’s just going to keep eating raisins and be like okay I don’t want M&Ms. This is what I’ve been eating, this tastes good.” Similarly, participants frequently select varieties of commonly used foods that are labeled as organic or “100% natural.” Many mothers gave their kids 100% fruit juice without dyes or artificial sweeteners, while other mothers selected organic bacon (“it’s less grease”) or organic frozen pizza bites. Mothers also stressed the need to control what others feed their children by preparing foods for visits to grandparents’ homes or times with babysitters and finding shortcuts to make using healthy foods easier (e.g., freezing fruit for smoothies).

Mothers in all groups described how their child wanted to eat the food from the mother’s plates. Because of this, mothers felt it was especially important they try to model healthy eating in front of their children. One mother described how her child did not eat certain foods until she started eating them: “She would not too much budge on eating vegetables and drinking water until I transitioned [to eating healthier].”

#### Use of "unhealthy" foods

Even so, many mothers admitted to including unhealthy foods in their kids’ diets as special treats, to save money and time, and for the family’s enjoyment. Mothers also admitted to occasionally using sweets or other foods as tools to keep kids calm or because their children liked them. For example, one participant shared: “I give her apple juice … and they told me [no] apple juice because of her new teeth coming in but she loves her some apple juice.” Furthermore, ease often trumped healthy eating for the mothers, with participants reporting that “a lot of times” they decided to cook something quick or pick up fast food, as this conversation shows:

*R4: But you know I would like to, you know say give my kid a salad, … like she said a vegetable, a starch and then you know chicken or something healthy. But you know sometimes with that you know with working a lot it’s hard to do all of that.*

*R6: Best laid plans for sure*.

Some mothers rarely use pre-packaged foods, while others used them often to save time and because they are easier. Some participants described mixes and precooked foods as cooking from scratch, so the actual consumption of processed foods might have been more common than reported. Furthermore, even mothers that previously stated they only served fresh and healthy foods, frequently used convenience foods like freeze-dried yogurt bits, Cheerios, or cereals puffs as snacks or to occupy children so they could get other things done.

#### Mealtime experiences

How mothers described meal experiences varied from generally calm to organized chaos, and participants try different techniques to make mealtime smoother (e.g., putting a child’s foods in separate bowls or allowing kids to have food from the mother’s plate). The majority of participants cooked meals for their children six days a week with one day off when they would eat pizza, leftovers, or some other prepared item. When situations allowed food choices for the child, the mothers provided young children parameters to help them make suitable choices. For example, a mother shared the following: “For the most part if it’s something we have and it’s something that is okay to eat then yes he gets to choose um but sometimes he wants you know a popsicle for breakfast so that’s obviously a no.”

Eating schedules were very structured for some families, while others described how every family member ate separately at different times of the day. Families with erratic schedules varied eating times substantially from day to day. The father’s schedule largely dictated whether a family ate together, whereby fathers who work at night or had different diet patterns ate when they wanted but if the father is home for dinner, everyone ate together. In all groups, mothers reported a shift away from requiring that kids clean their plates, instead allowing children to dictate the quantity they ate and not feeling concerned when children ate less food.

#### Introduction of complementary foods

While some mothers waited for the okay from their pediatrician or nutritionist to begin offering foods other than breastmilk or formula, other mothers made this decision on their own. One reason for choosing to supplement was the perceived need of the child to have certain items (e.g., two mothers gave their young infants water because “everybody need water”) or eat more (“crying for more and more”). Another reason for supplementing early was a feeling that the baby was advanced for his or her age: “My child he was already eating solid foods, beans, rice when he was two months old, for the fact that he was born with [front] teeth.” Some mothers decided to supplement their baby’s diet because they were excited or anxious to see how their child responded to the new foods or wanted to add more variety to their child’s diet. Several mothers let their child’s perceived desire for other foods guide them, starting supplemental foods when their baby showed interest in the mother’s food, “cued” them that it was time for other foods (“When that breast milk isn’t enough then I’ll be like okay [laughter] time for food”), or as a treat or a tool to calm their young baby (e.g., giving a baby under 1 year chocolate milk because she “loves it” and “she’s just quiet” when she has some).

### Drivers of early child feeding practices

**Fig. 1:**
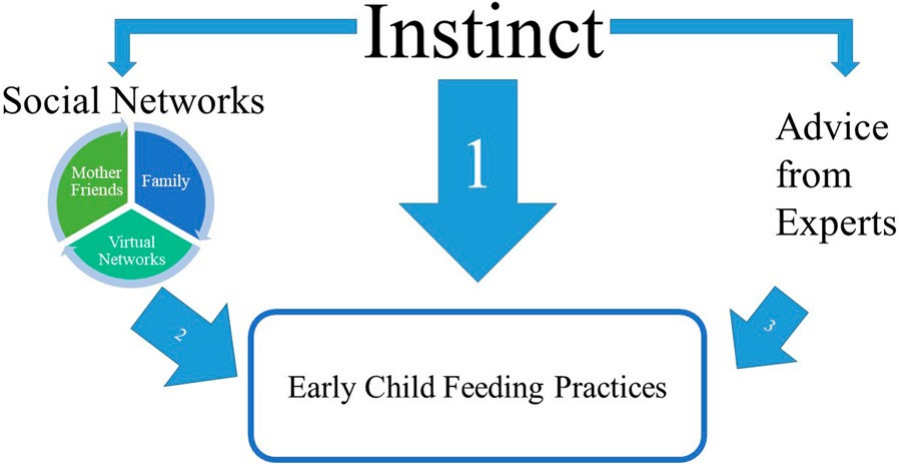
Major influencing factors on child feeding practices of mothers

Several factors influenced mothers' decisions about early childhood feeding (Fig. 1). Mothers explored the experiences of trusted sources but often relied on instincts to decide how and what to feed their children. As one mother said, “So I would just take a little from everybody. My thing is always, what my mother would always tell me when I was pregnant, listen to what your instincts tell you because that helps a lot more during parenting than what other people are saying.”

#### Maternal instincts

The strongest driver of early child feeding practices is mothers’ instincts about what is right for their child. As was highlighted in examples above (mothers giving infants water and the mother feeding solid foods to her two-month old), mothers felt strongly that they knew above everyone else what was right for their child, and they were willing to disregard expert advice that contradicted their views. Further, mothers were disinterested in advice that did not allow for exceptions because many believed their child to be that exception. Even for acute but minor health issues, mothers often gather information from others and the internet before or instead of talking to their doctor or taking a doctor’s advice. One mother described how she “didn’t like the answer my doctor, the precious doctor gave” regarding her daughter’s constipation “so I got home and done my own research and kind of fixed it on myself.” In another case, two mothers specifically ignored the doctor’s advice regarding the use of breastmilk as a treatment for pink eye, with one mother stating: “You have to be informed. I don’t think we just need to take evidence based medicine.”

#### Social networks

Mothers’ social networks include not only in-person contacts such as family and friends but also virtual communities. Participants valued the advice of other mothers (their mother, mothers in their peer-group) particularly. Many participants listed their mothers as a primary source for information on child feeding and child health, even though some participants felt their parents had some outdated views or practices and blamed their parents for spoiling their children by letting them eat whatever they wanted. Generally, participants valued personal experiences over expert advice, even for medical issues. For example, one mothers said, “You know when they tell you to give your baby this amount of Tylenol, you wanna know like from somebody else that gave their baby that amount of Tylenol. How’d that go? Were they allergic, you know was that too much? Not enough? Like I like personal opinions.”

Among parents of young children, online resources and digital social networks are a vital part of information gathering. Participants across groups reported belonging to private, invitation-only mothers' groups on Facebook and read articles on nutrition and childcare linked by friends or appearing on their Facebook feeds. For some participants, these online groups developed into in-person friendships. Other trusted sites for information on their child’s health include Babycenter (mentioned in three groups), Pinterest, WebMD, Mayo Clinic (each mentioned in two groups), and the CDC, the “American Association for Pediatrics” [sic, might mean the American Academy of Pediatrics], and individual pediatrician’s websites (discussed in one group).

Mothers use online resources when they have a question, want to learn more, need to find something out when the doctor’s office is closed, learn how things are done in other settings (e.g., in other states or outside the USA), research before a doctor’s visit, or discuss topics. Participants described doing a Google search first when researching a topic, calling Google their “best friend,” “the modern day Jesus,” and “good ‘ole doctor Google.” Mothers bookmarked their favorite sites and visited them repeatedly. Participants would not rely on just one online source, instead visiting multiple sites and supplementing that information with discussions with other mothers before making a decision. Participants also used online sources to provide justification for their opinions and decisions, “you just want that um security to make sure that okay I’m doing the right thing. You look it up and see how many other people agree or maybe there’s an article written on it or something.” Several mothers seemed to remember information gathered online better than what they are told by clinicians; for example, one mother learned to dilute juice from online blogs written by other mothers and “probably the doctor told me.” On the other hand, sometimes mothers felt pressure and guilt from social networks, particularly from online communities, for not always making healthy food choices, or what other community members think are the “right” healthy choices, for their children. As one mother said: “it’s like and you think doing so good and then they’re like well that’s not organic eggs.” A few participants even avoided online communities because they felt they were “judgy” and needed to “stay out of my business.”

Trust in online sources varied, although the majority of mothers reported being open to what they read online, trusting online friends “by instinct,” and not crosschecking things if they “seem plausible.” Many participants believed that the veracity of online information is based on the number of people agreeing with it and not by the source of the information. One mother described this as follows: “I gotta see it being consistent, and I have to like actually see how many people are visiting it, how many people were starring it, and stuff like that. Then I know what’s accurate, and what most people experience.” Although a few mothers reported double-checking “facts” by doing online research, across all four groups, only one participant reported trusting a website (cdc.gov) because the information was based on research.

#### Advice from experts

As described above, participants generally followed their instincts and are less or not likely to ask the pediatrician for advice about diet. For most participants, their doctor’s advice ranked behind that of mothers in their network, and women were split about if they would follow their doctor’s or their parent’s advice if they had opposite opinions about their child’s health. When asked specifically about child feeding, some mothers stated that they do not view nutrition to be a topic that needs to be discussed with their healthcare team: one mother stated she had not spoken to her child’s pediatrician about a food-related concern because “it’s something normal because a lot of kids don’t like vegetables so I it wouldn’t be something that would concern me.” Even among the participants who read and keep materials given to them by their doctor’s office, none spoke about returning to handouts for nutrition information, revisiting them only for developmental milestones and medication information. Several participants reported a reluctance to admit to their pediatrician when they did not follow their advice.

Even among mothers who preferred trusted medical sources like their pediatrician or a well-regarded local children’s hospital website, they still explored additional resources to find “alternatives” and get “second opinions.” Mothers followed a doctor’s advice most often when they had established trust with their pediatrician. This trust is usually developed over time or when the healthcare practitioner listens to the participant and respects her role as mother instead of the doctor being “more into their opinion than yours as a parent.” Participants particularly valued physician relationships where they “don’t feel wrong to ask anything.”

### Suggestions for improving patient resources

When providing feedback on existing handouts/pamphlets on early child feeding, mothers shared more about how advice from clinicians should and should not be presented. Participants were most receptive to messages that they agreed with, and they had the most negative comments when the advice contradicted their experiences, opinions, or expectations. For example, one mother shared: “they’re like don’t put cereal in the bottle so it’s like okay so what do you want me to do? Let my baby cry? …. my baby is like 3 or 4 months and I put cereal in his bottle and it worked.” Mothers were split between wanting to get as much information from their provider as possible (“I don’t think anybody is gonna complain about getting an extra sheet of paper if it’s about their baby.”) and feeling overwhelmed by the amount of handouts and advice provided (“I need to know what I’m supposed to do. What’s good for her, what’s not good for her. Like am I underfeeding her? Overfeeding her? Straight to the point.”). Mothers were particularly open to messages that target specific areas of concern.

Participants were very discouraged by negative language and the directive tone of some of the handouts: “I see ‘do not,’ ‘never,’ ‘do not,’ ‘never,’ ‘don’t,’ ‘don’t,’ ‘avoid,’ ‘avoid,’ it it seems very negative … It seems very strict.” Mothers felt that this negatively shut down discussion, while more positive messages encourage the mothers to talk openly with their clinicians, empower the mother, and do not undermine the mother’s role as gatekeeper for decisions around caring for their child. Mothers were often discouraged by the rigidness of the guidelines as well, because children develop differently and advice on feeding and other issues should reflect the developmental stage of their child and not just age. Guidelines also seemed capriciousness: “Um I guess she’s allowed to have milk today [on her baby’s birthday]… I don’t know two weeks ago if her stomach would be any different.”

## Discussion

In this study, mothers pregnant with their first child or with at least one child aged 0–2 years reported that proving healthy foods to their children was a priority and they were practicing many recommended practices for feeding young children; however, specific food choices and certain feeding behaviors showed that many women lacked a complete working knowledge of healthy diet choices or were unable or unwilling to consistently apply this knowledge. Feeding decisions were guided primarily by mothers’ instincts about what was appropriate for their child. Social networks, including both in-person relationships and digital communities, were the next most used source for child feeding information. Clinicians were approached least often for guidance. Finally, women shared that healthcare messaging should have a positive tone, reflect flexibility and personalization of expert recommendations, and address areas of concern.

Participants reported adherence to many early dietary and child feeding behaviors that have been shown to be beneficial, for example avoiding controlling food practices like forcing a child to “clean her plate” and modeling healthy eating. Although acting with the best of intentions, mothers’ actions show an incomplete knowledge of nutrition recommendations. Mothers used food as tools for calming their child and assumed heathy sounding foods were always nutritious (swapping to organic foods but still selecting high fat, high sodium items, 100% fruit juices, freeze-dried yogurt bites, cereal puffs). These results support quantitative data showing that even though there has been some improvements overall, there is still substantial work needed to optimizing early child nutrition [9].

Mothers sometimes used less healthy or not recommended foods as tools to decrease the stressfulness of mealtimes or childcare; mothers shared instances where they used sugary snacks to distract a child or added cereal to a young infant’s bottle to increase sleep. These finding support other work where mothers report that both general child feeding practices [10] and transitioning to solid foods [11] were driven more by short term outcomes (e.g., child eating a meal) than long-term ones (e.g., avoiding childhood obesity). Similarly, although mothers believed family meals are important for forming healthy eating behaviors in small children, whether a family ate together was largely driven by the father’s schedule. This has been shown here and in other studies [10, 12], even though mothers drove food choices in their families. Involving partners might be key to maximize early child feeding practices.

Women prefer to gather information on early feeding from a variety of sources, but they are most influenced by their own instincts. Participants felt that only they had all the information needed to know what was best for their child. This is similar to the results reported in a qualitative study of infant feeding behaviors in Australian mothers with low education, where mothers reported that when deciding when and how to transition to solid foods they listen to advice but make up their own mind and that “mother knows best.” [13].

Participants reported that they valued personal experiences over expert advice, one participant going as far as stating she did not think mothers needed to rely on “evidence based medicine” to make decisions. Mothers used a wide social network to gather the personal experiences and stories needed to make decisions around early child feeding. Participants cultivated online communities to gather information, find support, and make decisions. This reliance on social media for child health information has been reported elsewhere [13–15]. In this study, participants used their instincts to decide if virtual friends or online sources, often identified through internet searches or social media links, were trustworthy, with response popularity being a good indicator of veracity. Tellingly, only one participant said she trusted an online source because it was backed by research. In a study of internet use and vaccine views [16], parental internet use was inversely associated with scientifically supported beliefs bout vaccinations. Given the high reliance on the internet among mothers in this study, future research is needed to understand if this relationship is consistent for childhood feeding.

The ways social networks were used by these mothers differed from another qualitative study of early child feeding. In Spence et al. [10] mothers primarily used other mothers’ behaviors as examples of how they did not want to feed their child and reported that types of foods they consumed in childhood influenced feeding decisions for their children. In this study, however, participants’ use of social networks was more focused on copying behaviors the mother agreed with, and their upbringing was not an important influencer of diet choice.

Mothers in this and other studies [10, 13] report that doctors are a less used, and in some cases less valued, source of information on early child feeding. In this study, mothers reported several reasons for this including: (1) clinicians did not respect their instincts and opinions; (2) child feeding challenges were normal parts of everyday life and not a medical issue; and (3) not having a good patient-provider relationship. Conversely, when mothers had an established relationship with their child’s doctor with open communication, mothers said they were more likely to listen to and heed health advice.

Similarly, participants were most receptive to patient resources and advice when the tone was open and positive and valued their role as mother. Expert guidelines for early child feeding seemed overly rigid and capricious, and participants wanted more flexibility and personalization to better suit their unique child. These findings align with the opinions expressed by mothers in other studies of early child feeding [13, 17–20]. Women reported that negative language and lists of things to avoid/not do were major turn offs and shut down lines of communication, while more positive messaging was more likely to encourage them to discuss things with their clinicians.

These data provides important guidance on how clinicians should share expert recommendations for early child feeding with patients. Since mothers were particularly open to messaging targeting areas of concern or with which they agreed, clinicians should use evidence-based methods such as motivational interviewing [21] to identify areas of concern and use mothers’ questions as a starting point for discussing issues that might be more contentious. Mothers respond best when they have open communication with positive messaging, and clinicians should work on building relationships with parents and prioritize honest, collaborative discussions. Furthermore, given that some mothers may be reluctant to share honestly when they did not follow guidelines, it is important for providers to probe and ask specifically about early child feeding practices to identify and address issues of concern. Finally, well-done handouts and websites can be a way to open up conversations, provide key information, and help a mother-clinician team find areas needing further discussion or clarification. In the Melbourne Infant Feeding Activity and Nutrition Trial [10], the most discussed source of health information was the program, showing that materials based on expert advice, when delivered well, can be a powerful source of information on early child feeding.

This study has several limitations. Combining mothers during their first pregnancy, with a single child under 2 years, and with multiple children compromised our ability to tease out nuances between these groups. Similarly, this study did not account for the weight of the mothers’ children, and previous work has shown differences in early child feeding patterns by parents of children who were normal weight or overweight [22]. Finally, additional focus group discussions might have provided more clarity and nuance around these issues. The overall consistency between groups (attempted stratifications did not results in differences in key themes or interpretations indicating that meaning saturation might have been reached) and guidance showing that four focus groups is sufficient for reaching code saturation [23] indicate that this is unlikely to be an important limitation.

## Conclusions

Research exploring mothers’ experiences with, feelings around, influences of, and challenges around early child feeding is needed [9, 12], and this study helps to fill this gap. We report that pregnant women and mothers with at least one child aged 0–2 years prioritized a healthy diet for their children; however, they applied expert recommendations inconsistently or in a way that reflects only a partial understanding of optimal child feeding practices. Decisions were guided primarily by the mothers’ instinctual feelings about what is best for their child, although they did use online and in-person social networks to gather information on child feeding. Clinicians were used least often as a source of information on child feeding, but positive, supportive messaging from trusted medical sources was welcome.

Clinicians can use these finding to assist parents in making decisions about early childhood feeding. Given the high prevalence of childhood overweight and obesity, the serious short and long-term physical and mental toll of excess weight in childhood, and the importance of early childhood feeding practices on long-term health, body composition, and eating behaviors, it is vital to identify the best ways to work with parents and support their efforts to feed their children well.

## Data Availability

The datasets used and/or analyzed during the current study are available from the corresponding author on reasonable request.
